# Utility of 15(S)-HETE as a Serological Marker for Eosinophilic Esophagitis

**DOI:** 10.1038/s41598-018-32944-8

**Published:** 2018-09-28

**Authors:** Shaolei Lu, Michael Herzlinger, Weibiao Cao, Lelia Noble, Dongfang Yang, Jason Shapiro, Jonathan Kurtis, Neal LeLeiko, Murray Resnick

**Affiliations:** 1 Department of Pathology and Laboratory Medicine, Rhode Island Hospital, Warren Alpert Medical School of Brown University, Providence, USA; 2 Division of Pediatric Gastroenterology, Nutrition, and Liver Diseases, Hasbro Children’s Hospital, Warren Alpert Medical School of Brown University, Providence, USA

## Abstract

The pathogenesis of eosinophilic esophagitis (EoE) involves Th2-mediated eosinophil recruitment and degranulation into the esophagus. However, measuring serum Th2 cytokines, eosinophils, and eosinophil-derived products does not reliably distinguish EoE from control populations. Non-invasive methods to diagnose EoE are lacking. We evaluated the diagnostic value of a novel candidate biomarker of EoE: 15(S)-hydroxyeicosatetraenoic acid (HETE). We used immunoassay to measure 15(S)-HETE and cytokine profiles in patients undergoing endoscopy with known or suspected EoE. 31 subjects were enrolled, 16 with EoE, and 15 with an alternate diagnosis. 15(S)-HETE was elevated in the EoE group compared to non-EoE group. The sensitivity and specificity of 15(S)-HETE to be used as a non-invasive marker is 50% and 80%, respectively. 15(S)-HETE may aid in the diagnosis of EoE.

## Introduction

Eosinophilic esophagitis (EoE) is a chronic, allergen-mediated, inflammatory condition affecting the esophagus^1^. Its prevalence has been increasing, and has been recently estimated at 56.7/100,000^2^. In teenagers and adults, EoE typically presents with dysphagia, heartburn, or food impaction, whereas toddlers and young children tend to present with nonspecific symptoms such as feeding intolerance, growth failure, or abdominal pain.

Diagnosis of EoE in all age groups is complicated by clinical and histologic overlap with other gastrointestinal conditions, particularly gastroesophageal reflux disease (GERD). Distinguishing these disorders is invasive, expensive, and time-consuming. Once a diagnosis of EoE is established, monitoring treatment efficacy poses similar difficulties. There is little correlation between symptom severity and esophageal inflammation, so endoscopy is required^3^. Patients may benefit from reliable and non-invasive methods to diagnosis and monitor EoE.

Efforts have usually focused on identifying peripheral blood markers of EoE. A subset of EoE patients have increased peripheral absolute eosinophilia counts (AEC), compared to controls^3,4^, though AEC alone does not reliably differentiate these populations. Eosinophil-specific mediators, such as eosinophil-derived neurotoxin and eotaxin-3 are increased in the plasma of EoE subjects with active disease, and correlate with eosinophil infiltration into the esophagus^4^. EoE is recognized as a Th2-predominant systemic inflammatory process. Th2 cytokines such as IL-5 and IL-13 are increased in the plasma of EoE subjects compared to controls^5–8^. While these studies provide details about the pathogenesis of EoE, none have identified a simple blood test which can reliably diagnose EoE. Endoscopy with biopsy remains the gold standard for diagnosing and monitoring EoE disease activity.

Recent evidence suggested a role for arachidonate 15-lipooxygenase (ALOX15) in EoE. ALOX15 is up-regulated in esophageal mucosal biopsies of EoE patients compared to GERD patients and healthy controls^9–11^. Similarly, esophageal squamous epithelial cells of EoE patients, but not controls, are diffusely positive on immunostaining for ALOX15^10^. Staining for ALOX15 may be helpful in equivocal cases of esophageal eosinophilia, where the diagnosis of EoE is unclear^12^. ALOX15 is mainly expressed in cytosol and plasma membrane in squamous cells of EoE mucosa^10^ and bronchial epithelial cells after IL-13 exposure^13^. So far, there is no evidence demonstrating that ALOX15 can be secreted into circulation. However, 15(S)-hydroxyeicosatetraenoic acid (15(S)-HETE), a metabolite of ALOX15 and detectable in peripheral blood^14^, could be a good indicator of EoE presence and/or severity. Detectable 15(S)-HETE have been shown in the urine and its diagnostic utility was proposed for patients with Zellwegar syndrome^15^. 15(S)-HETE appeared to be involved in asthma, an atopic condition like EoE^16^. Severe asthmatics had higher levels of 15(S)-HETE in bronchoalveolar lavage samples compared to mild or moderate asthmatics and healthy controls^17^.

We hypothesized that (1) 15(S)-HETE would be elevated in the sera of EoE patients compared to those without EoE; (2) that 15(S)-HETE, in combination with peripheral eosinophil count and Th2 cytokines typically found in EoE, reliably distinguish EoE from other conditions.

## Materials and Methods

The study design was reviewed and approved by Investigational Review Board (IRB) of Lifespan/Rhode Island Hospital (Providence, RI). All methods were performed in accordance with the relevant guidelines and regulations. Written informed consent was obtained from all participants and/or their legal guardians.

### Subject selection

Subjects were recruited from the Division of Pediatric Gastroenterology at Hasbro Children’s Hospital, Warren Alpert Medical school of Brown University (Providence, RI), a tertiary care center located in an urban setting. Over a 12-month period, patients undergoing upper endoscopy with known or suspected EoE were screened.

Inclusion criteria included undiagnosed children with symptoms and medical history concerning for EoE. Symptoms considered suspicious for EoE included epigastric abdominal pain, heartburn, dysphagia, food impaction, vomiting, or failure to thrive. We also included known EoE patients who had not been treated with topical or systemic steroids during the 8 weeks prior to endoscopy, and those on dietary-elimination regimens for whom food re-introduction endoscopy was being performed. Exclusion criteria included patients with comorbid gastrointestinal or autoimmune diseases such as inflammatory bowel disease, celiac disease, Helicobacter gastritis, and thyroid disease.

We enrolled 31 participants. Patients or their legal guardians provided written informed consent. Pertinent medical information including demographics, concomitant medications, family and participant medical history was collected.

Participants were categorized into two groups: (1) EoE or (2) non-EoE. Diagnosis of EoE was made according to 2013 census guidelines^1^. This included at least 8 weeks of proton-pump-inhibitor (PPI) administration prior a diagnostic endoscopy to exclude PPI-responsiveness. In instances in which PPI responsiveness was not evaluated (N = 5), EoE diagnosis was supported by the presence of the clinical and pathologic findings typical for this condition.

### Peripheral Absolute Eosinophil Counts (AECs) and Cytokine and15(S)-HETE immunoassays

Peripheral blood was drawn from each participate just prior to endoscopy. A routine hematology complete blood count (CBC) was performed on each sample. The absolute eosinophil count (AEC) was extracted from the CBC report. The remaining plasma was immediately aliquoted and kept in −70C for batch immunoassays. Assays were performed in triplicate and a standard curved was generated for each analyte. Cytokine assays were carried out on BioPlex 200 (Luminex, Austin TX), a multiplex bead-based immunoassay testing platform using a custom made 11-plex sandwich capture assay kit developed previously^18^. IL-1, IL-2, IL-4, IL-5, IL-6, IL-8, IL-10, IL-12, IL-13, gamma interferon (IFN-γ), and tumor necrosis factor alpha (TNF-α) were the cytokines tested.

15(S)-HETE assay was carried out on a Synergy™ H4 Hybrid Microplate Reader (BioTek, Winooski, VT) using an 15(S)-HETE EIA Kit (Item No. 534721) from Cayman Chemical Company (Ann Arbor, MI) following manufactory protocol. Included in the Kits were plates pre-coated with mouse anti-rabbit IgG, a rabbit polyclonal antibody specific to 15(s)-HETE and a 500 ng/ml standard based upon which standard curves were generated to quantitate individual sample. The detect limit was approximately 170 pg/ml. Linear range was from 170–5000 pg/ml. Samples whose original concentrations falling out of the higher end of the linear range was diluted and re-assayed. The wavelength to detect the absorbance was set at 410 nm.

### Endoscopic Examination and Histological Evaluation of Endoscopic Biopsies

A standard upper endoscopy on was performed on each patient. At minimum, two biopsies from the proximal and distal esophagus were obtained for the study. Per routine practice, biopsies were also obtained from duodenum and stomach to exclude other gastrointestinal pathology. Histological assessments were conducted by the study pathologists (SL, MR) to determine eosinophil counts. The maximal eosinophil count in one high power field from proximal and distal biopsies was used in this study for each patient.

### Statistical analysis

Data were analyzed with JMP Pro 13.0 (SAS Institute, Cary, NC). Student’s t-test was used to compare continuous variables (two-tail). Chi-square test was used to compare categorical variables and when the cell numbers were small, Fisher’s exact test was used. Receiver operating characteristic (ROC) analysis was performed based on logistic regression modeling. Cut-off value for each parameter was based on the threshold level in logistic regression when the sum of sensitivity and specificity was the largest. Spearman correlation was used to evaluate association between two continuous variables. Results were considered significant for P values less than 0.05.

## Results

### Subject characteristics

A total of 16 EoE patients and 15 non-EoE patients were enrolled in the study. Patient characteristics and their correlations with EoE status were summarized in Table 1. The non-EoE patients included 10 cases of functional dyspepsia, 3 cases of GERD, and 2 cases of globus. There was no age difference between EoE and non-EoE patients. There was a male predominance in EoE patients (73.68%). Symptomatically, food impaction was reported by EoE patients more often than non-EoE patients (25% vs 0%, p = 0.1012), while non-EoE patients reported more heartburn (53.3% vs 25%, p = 0.1030). Endoscopically, presence of furrows was significantly more frequently observed in EoE patients than in non-EoE patients (93.8% vs 26.7%, p = 0.0002) and overall abnormal findings were significantly higher in EoE patients than in non-EoE patients (93.8% vs 40%; p = 0.0021). EoE patients reported significantly more atopy than non-EoE patients (93.8% vs 60.0%, p = 0.0373). Microscopically, biopsies from EoE patients had significantly higher intraepithelial eosinophil densities than those from non-EoE patients (49.8/hpf vs 2.6/hpf, p < 0.0001). EoE biopsies more commonly had basal cell hyperplasia and subepithelial fibrosis (p = 0.0373 and 0.0068, respectively).

**Table 1:** Patient characteristics and serological analyte levels.

Clinical characteristics and analytes	EoE (n = 16)	Non-EoE (n = 15)*	P-value**
Mean age at biopsy (±SEM)	11.2 ± 1.3	12.1 ± 1.3	0.6641
Male subjects (n, %)	14 (73.68)	5 (26.32%)	**0**.**0014**
Symptoms			
Vomiting (n, %)	5 (31.3)	6 (40.0)	0.6107
Dysphagia (n, %)	10 (62.5)	8 (53.3)	0.3076
Abdominal pain (n, %)	7 (43.8)	11 (73.3)	0.0921
Food impaction (n, %)	4 (25.0)	0 (0.0)	0.1012^F^
Heartburn (n, %)	4 (25.0)	8 (53.3)	0.1030
Failure to thrive (n, %)	2 (12.5)	3 (20.0)	0.5698
Abnormal EGD findings (n, %)	15 (93.8)	6 (40.0)	**0**.**0021**^F^
Esophageal erythema (n, %)	4 (24.0)	3 (20.0)	0.7389
Rings (n, %)	2 (12.5)	0 (0.0)	0.4839^F^
Ridging (n, %)	3 (18.8)	1 (6.7)	0.3052^F^
Furrows (n, %)	15 (93.8)	4 (26.7)	**0**.**0002**^F^
White exudates (n, %)	7 (43.8)	2 (13.3)	0.0564
Erosions (n, %)	5 (31.3)	2 (13.3)	0.2265
Presence of any atopy (n, %)	15 (93.8)	9 (60.0)	**0**.**0373**^**F**^
Rhinitis (n, %)	11 (68.8)	7 (46.7)	0.2113
Dermatitis (n, %)	6 (37.5)	4 (26.7)	0.5179
Food allergy (n, %)	9 (56.3)	3 (20.0)	0.0659^F^
Asthma (n, %)	8 (50.0)	4 (26.7)	0.1794
Histological findings			
Max eosinophil count*** (±SEM)	49.8 ± 6.4	0.8 ± 0.5	**<0**.**0001**
Basal cell hyperplasia (n, %)	7 (43.8)	1 (6.7)	**0**.**0373**^F^
Submucosal fibrosis (n, %)	7 (43.8)	0 (0.0)	**0**.**0068**^F^
HETE (pg/ml, ± SEM)	13782.8 ± 4541	5755.9 ± 915	0.2131^W^
AEC (K/µl, ± SEM)	0.52 ± 0.15	0.17 ± 0.032	**0**.**0291**
Th2 cytokines (pg/ml, ± SEM)			
IL-4	632.5 ± 213.9	229.4 ± 74.8	0.0967^W^
IL-5	59.1 ± 17.7	17.4 ± 8.7	0.0583^W^
IL-6	218.1 ± 76.8	53.4 ± 24.7	0.0587^W^
IL-10	230.8 ± 62.8	67.4 ± 28.1	**0**.**0311**^W^
IL-13	523.3 ± 156.6	218.4 ± 65.3	0.1047^W^
Other cytokines (pg/ml, ±SEM)			
IL-1	523.0 ± 142.2	270.6 ± 89.1	0.2430^W^
IL-2	63.2 ± 37.3	19.3 ± 16.4	0.2666^W^
IL-8	7.7 ± 3.7	3.2 ± 0.84	0.3684^W^
IL-12	523.6 ± 249.3	113.9 ± 49.5	0.1586^W^
TNF-α	297.9 ± 79.8	136.0 ± 42.4	0.1982^W^
IFN-γ	2017.3 ± 696.2	1060.0 ± 386.4	0.2165^W^

EoE: eosinophilic esophagitis; SEM: standard error of mean; EGD: esophagogastroduodenoscopy.

*Non-EoE patients include 10 cases of functional dyspepsia, 4 cases of GERD, and 2 cases of globus.

**P-value: 2-tail t-test for continuous variables; “W” indicates Wilcoxon nonparametric test if the data distribution is not Gaussian; chi-square for categorical variables; “F” indicates 2-tail Fisher’s exact test when the cell number is too small for chi-square.

***Maximal eosinophil count was determined from the high-power field with the highest count.

### Peripheral 15(S)-HETE, absolute eosinophil count, and cytokines in EoE and non-EoE subjects

**Figure 1:**
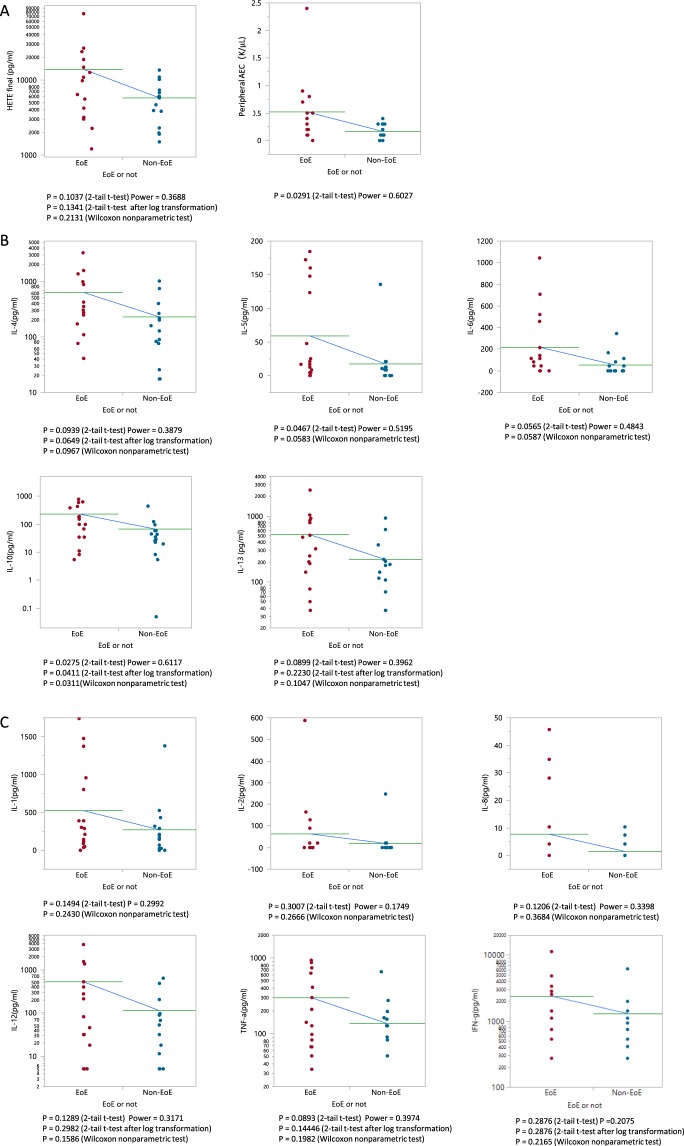
(**A**) Peripheral 15(S)-HETE level and absolute eosinophil count (AEC) in EoE and non-EoE patients. (**B**) Peripheral Th2 cytokine levels in EoE and non-EoE patients. (**C**) Peripheral non-Th2 cytokine levels in EoE and non-EoE patients. Horizontal line indicates level of mean. Y-axis is in log-scale if a normal distribution is achieved after log-transformation.

A 2.4-fold elevation of peripheral 15(S)-HETE level was observed in EoE patients (p = 0.2131; Table 1 and Fig. 1A). There were significant increases of peripheral absolute eosinophil count in EoE patients compared to non-EoE patients (3.1-fold; p = 0.0291; Table 1 and Fig. 1A). However, 9 out of 16 EoE patients and 15 out of 15 non-EoE patients had normal AEC based on a reference range of 0.0–0.5 K/µl.

Peripheral Th2-related cytokine elevations (2.4–4.1-fold; p = 0.0311–0.1047) were observed in EoE patients compared to non-EoE patients (Fig. 1B) including IL-10 significantly elevated in EoE patients (3.4-fold; p = 0.0311). Of the cytokines not directly involving Th2 activation, the fold changes ranged from 1.9 to 4.6 (p = 0.1586–0.3584; Table 1 and Fig. 1C).

While no significant association existed between 15(S)-HETE and peripheral AEC (Spearman ρ = 0.3306, p = 0.744), AEC was significantly associated with all the Th2 cytokines, IL-12 and TNF-α (Table 2). All cytokines were not significantly associated with 15(S)-HETE (Table 2).

**Table 2:** Spearman correlation of 15(S)-HETE and peripheral AEC with cytokines.

Correlation	15(S)-HETE		Peripheral AEC	
	Spearman ρ	P-value	Spearman ρ	P-value
15(S)-HETE	N.A.	N.A.	0.3306	0.0744
Peak eosinophils count in esophagus	0.3635	**0**.**0444**	0.6272	**0**.**0002**
IL-4*	0.2725	0.1381	0.4495	**0**.**0127**
IL-5*	0.2492	0.1763	0.4304	**0**.**0176**
IL-6*	0.1916	0.3019	0.4507	**0**.**0124**
IL-10*	0.276	0.1328	0.4135	**0**.**0231**
IL-13*	0.2452	0.1836	0.4	**0**.**0285**
IL-1	0.1958	0.2911	0.3487	0.0589
IL-2	0.3035	0.097	0.3436	0.063
IL-8	0.1098	0.5564	0.2388	0.2038
IL-12	0.2903	0.1132	0.4592	**0**.**0107**
TNF-α	0.2562	0.1641	0.4439	**0**.**014**
IFN-γ	0.2341	0.205	0.2991	0.1083

*Cytokines involving Th2 pathway.

### Peripheral 15(S)-HETE levels and tissue histology in EoE and non-EoE patients

We assessed the relationship between peripheral 15(S)-HETE levels and histologic characteristics of esophageal biopsies. The correlation between 15(S)-HETE levels and peak eosinophil count was significant (Spearman σ = 0.3635; p = 0.0444; Table 2). There was significant difference in 15(S)-HETE levels among participants with esophageal fibrosis (P = 0.0074) while no difference was seen with respect to basal cell hyperplasia (P = 0.6569).

### Utilities of 15(S)-HETE, absolute eosinophil count and cytokines in EoE diagnosis

**Figure 2:**
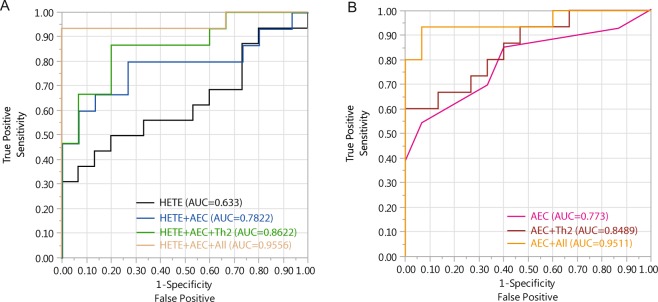
Receiver operating characteristic (ROC) curves for diagnosis of EoE. (**A**) The area under curve (AUC) was calculated for 4 conditions: 15(S)-HETE alone; combination of 15(S)-HETE, and peripheral AEC; combination of 15(S)-HETE, AEC and Th2 cytokines (IL-4, IL-5, IL-6, IL-10, and IL-13); combination of 15(S)-HETE, AEC, and all the cytokines. (**B**) The AUC was calculated for 3 conditions: Peripheral AEC alone; combination of AEC and Th2 cytokines; combination of AEC and all the cytokines.

Receiver operating characteristic (ROC) curves were constructed to investigate the use of peripheral 15(S)-HETE, AEC and cytokines. 15(S)-HETE alone yielded an area under curve (AUC) of 0.633 for the diagnosis of EoE with sensitivity of 50%, specificity of 80% and accuracy rate of 64.5% in this cohort when the cut-off value was set at 7500 pg/ml (Table 3 and Fig. 2). AEC alone yielded an AUC of 0.773 for diagnosis of EoE with sensitivity of 53.3%, specificity of 93.3% and accuracy rate of 71% with cut-off value set at 0.4 K/µl. Individual cytokines related to Th2 activation yielded AUC values ranging from 0.671 to 0.7292 with sensitivities from 43.8% to 62.5%, specificities from 80% to 93.3%, and accuracy rates from 64.5% to 74.2% (Table 3). When 15(S)-HETE and AEC were combined in one model, the AUC increased to 0.7822. When AEC and Th2 cytokines were combined, the AUC became 0.8489. Adding 15(S)-HETE to AEC and Th2 cytokines improved the AUC to 0.8622 (Fig. 2A,B). When AEC and all the cytokines were combined, the AUC was 0.9511; addition of 15(S)-HETE improved the AUC to 0.9556 (Fig. 2A,B).

**Table 3:** Areas under curve (AUC), cut-off values, specificities and sensitivities of assays to diagnose EoE.

	AUC	Cut-off value*	Sensitivity	Specificity	Accuracy **	P-value***
15(S)-HETE	0.633	≥7500	50.0%	80.0%	64.5%	0.0340
AEC	0.773	≥0.4 K/µl	53.3%	93.3%	71.0%	0.0024
IL-4	0.675	≥270	56.3%	80%	67.7%	0.0527
IL-5	0.700	≥22	43.8%	93.3%	67.7%	0.0338
IL-6	0.6917	≥90	50%	80%	64.5%	0.0295
IL-10	0.7292	≥96	62.5%	86.7%	74.2%	0.0159
IL-13	0.671	≥230	56.3%	80%	67.7%	0.0518
IL-12	0.648	≥210	43.8%	86.7%	64.5%	0.0600
TNF-a	0.635	≥280	37.5%	86.7%	64.5%	0.0707

*The unit of analytes is pg/ml except AEC which is K/µl.

**Accuracy is the summation of true positive and true negative.

***Whole Model Test in logistic Fit analysis.

## Discussion

Diagnosis and management of EoE is complicated by a lack of reliable peripheral biomarkers of disease activity. Our goal was to determine if plasma 15(S)-HETE, a downstream metabolite of ALOX15, can be used as noninvasive markers for EoE. We hypothesized that 15(S)-HETE, in combination with Th2 cytokines, may distinguish EoE from non-EoE patients.

We found that 15(S)-HETE levels were increased 2.4-fold in the plasma of the EoE group compared to the non-EoE controls. To our knowledge, this finding has not been previously reported in the literature. Its significance is however unclear, although we speculate that 15(S)-HETE may play a role in the pathogenesis of EoE. There is conflicting evidence on whether 15(S)-HETE exhibits pro- or anti-inflammatory properties. 15(S)-HETE and related proteins may contribute to the resolution of inflammation by (1) stimulating macrophages to remove apoptotic cells, and (2) preventing fibroblasts from forming collagen and remodeling tissue^19–21^. Other studies have suggested a pro-inflammatory/pro-fibrotic role in diseases such as atherosclerosis and rheumatoid arthritis^22,23^. 15(S)-HETE is increased in asthmatics compared to controls, and correlates with disease severity^17^. Perhaps in the acute setting, 15(S)-HETE, contributes to the resolution of inflammation, whereas its presence in conditions with prolonged inflammation, as seen in EoE^20^, may just reflect chronicity.

We assessed the usefulness of 15(S)-HETE as a clinical tool. When we compared serum 15(S)-HETE values to esophageal histology, we found no significant correlation between 15(S)-HETE and maximum eosinophil count (R^2^ = 0.128). Likewise, serum 15(S)-HETE levels are no different in participants whose esophageal biopsies demonstrated subepithelial fibrosis or basal cell hyperplasia. We found that 15(S)-HETE levels did not adequately distinguish EoE from non-EoE cases with AUC of 0.633. Given these findings, 15(S)-HETE alone is not a useful marker of esophageal inflammation in EoE and is insufficiently sensitive and specific to be used as a non-invasive diagnostic measure.

In keeping with prior studies, which characterize EoE as a Th2 predominant process, we showed an increase in all peripheral Th2-related cytokines in the EoE compared to non-EoE groups. The accuracy of these cytokines in determining a diagnosis of EoE was low (approximately 70%), comparable to previously reported accuracy values^8^. When we combined our novel marker with AEC and Th2 cytokines, AUC improved to 0.7822 (15(S)-HETE and AEC) and 0.8622 (15(S)-HETE, AEC and Th2 cytokines). The combination of 15(S)-HETE, peripheral AEC, and all cytokines reached a maximal AUC of 0.9556 (Fig. 2A), a little higher than when AEC and all cytokines combined (AUC of 0.9511) (Fig. 2B).

In our study, Th2 cytokines IL-10 correlated with levels (p = 0.0311). IL-5 and IL-6, also members of Th2 cytokine, had weak correlation with 15(S)-HETE (p = 0.0583 and 0.0587, respectively). The relationship between these proteins is unclear, as it pertains to the pathogenesis of EoE. Th2 cytokines have been shown to stimulate ALOX15 activity^16,19^. In EoE, we suspect that Th2 cytokines mobilize ALOX15-expressing cells, which in turn generate 15(S)-HETE. We found a significant correlation between AEC and 15(S)-HETE. As such, we presume eosinophils are primarily responsible for the pool of circulating 15(S)-HETE. Since Th2 cytokines also induce expression of ALOX15 in mast cells, monocytes, and epithelial cells, it is likely that these contribute as well^5,24–26^.

Serum IL-1, IL2, IL-8, IL-12, TNF-α and IFN-γ, typically associated with Th1 activation, were markedly elevated in the EoE group although statistical significance was not obtained. Th1 activation in esophageal biopsies of EoE subjects has been shown in previous studies^5,27,28^. However, our finding of these TH1 cytokines in the serum of EoE patients is novel. In mice, HETE proteins have been shown to stimulate macrophage production of TNF-α^29^. Similarly, human studies demonstrate increased mononuclear cell TNF-α production among participants with variations in HETE-related genes^30^. Together, these findings suggest a relationship among 15(S)-HETE and mononuclear cell production of Th1 cytokines in the pathogenesis of EoE. The participation of Th1 pathway in EoE is consistent with our AUC study where AUC values increased if all the cytokine data were incorporated in the analysis compared with Th2 cytokines alone.

There are limitations in our study. Since there is no gold standard to diagnose EoE, it is possible that we misclassified some of our enrollees. PPI-responsiveness in 5 of the 15 subjects in the EoE group was not assessed endoscopically due to insufficient follow-up or parent preference. Furthermore, majority of the patients in both groups were taking PPI at the time of endoscopy, generally at a dose of at least 1 mg/kg/day for younger patients or 30–40 mg/day for older patients. Given recent evidence suggesting anti-inflammatory properties of PPIs, independent of acid suppressive mechanism, it is unclear how the concurrent administration of this medication may influence our findings^31^. Nevertheless, we found no correlation between 15(S)-HETE levels and PPI exposure at the time of blood sampling (Supplemental Table 1).

Testing peripheral 15(S)-HETE levels should be limited to the patients who are clinically suspicious for EoE. 15(S)-HETE levels were not statistically different in patients with or without specific symptoms listed in Table 1 (data not shown). Therefore, 15(S)-HETE should not be used to triage patients with general symptoms such as abdominal pain. Utilities of the assay in diagnosing diseases that have similar symptoms such as celiac disease and inflammatory bowel disease are to be investigated in future studies.

Previous work has shown atopic status as an independent predictor of serum Th2 cytokine levels, regardless of participant group (i.e. EoE vs non-EoE control)^8,32^. In contrast, in our study, Th2 cytokine profiles and 15(S)-HETE levels corresponded to diagnosis and not to atopic status (Supplemental Table 2). Finally, the diagnosis of atopy was based on patient/parent reporting and/or supporting physician documentation. We did not employ IgE levels, radioallergosorbent test (RAST), skin, or patch testing to confirm. Thus, concern remains for atopy as a confounding variable, particularly since EoE and atopic conditions are frequently coincident.

In the current study, peak eosinophil count (PEC) was used to as an index to quantitate the severity of the EoE pathology and it was significantly associated with 15(S)-HETE and peripheral AEC (Table 2). Recently studies have shown that a comprehensive EoE histology scoring system (EoEHSS) was better than PEC for disease diagnosis and monitoring^33,34^. With EoEHSS gaining more acceptance, further EoE studies are likely to adopt EoEHSS as a standard histological evaluation.

In conclusion, 15(S)-HETE may participate in the dysregulated immune response which characterizes EoE. Further studies are needed to clarify its role in this process. 15(S)-HETE alone is no better than AEC and Th2 cytokines as a noninvasive means to distinguish EoE from other GI conditions. However, there may be role for this novel marker in combination with other peripheral markers, such as eosinophil-derived neurotoxin (EDN), eotaxin-3, and IL-13, in the diagnosis and management of EoE.

## Electronic supplementary material

Supplemental tables

## References

[1] Dellon ES (2013). ACG clinical guideline: Evidenced based approach to the diagnosis and management of esophageal eosinophilia and eosinophilic esophagitis (EoE). Am J Gastroenterol..

[2] Dellon ES (2014). Epidemiology of eosinophilic esophagitis. Gastroenterol Clin North Am..

[3] Gupta SK (2008). Noninvasive markers of eosinophilic esophagitis. Gastrointest Endosc Clin N Am..

[4] Konikoff MR (2006). Potential of blood eosinophils, eosinophil-derived neurotoxin, and eotaxin-3 as biomarkers of eosinophilic esophagitis. Clin Gastroenterol Hepatol..

[5] Straumann A, Bauer M, Fischer B, Blaser K, Simon HU (2001). Idiopathic eosinophilic esophagitis is associated with a T(H)2-type allergic inflammatory response. J Allergy Clin Immunol..

[6] Straumann A (2005). Cytokine expression in healthy and inflamed mucosa: probing the role of eosinophils in the digestive tract. Inflamm Bowel Dis..

[7] Yamazaki K (2006). Allergen-specific *in vitro* cytokine production in adult patients with eosinophilic esophagitis. Dig Dis Sci..

[8] Blanchard C (2011). A striking local esophageal cytokine expression profile in eosinophilic esophagitis. J Allergy Clin Immunol..

[9] Blanchard C (2007). IL-13 involvement in eosinophilic esophagitis: transcriptome analysis and reversibility with glucocorticoids. J Allergy Clin Immunol..

[10] Matoso A (2013). Expression microarray analysis identifies novel epithelial-derived protein markers in eosinophilic esophagitis. Mod Pathol..

[11] Wen T (2013). Molecular diagnosis of eosinophilic esophagitis by gene expression profiling. Gastroenterology..

[12] Matoso A (2014). Correlation of ALOX15 expression with eosinophilic or reflux esophagitis in a cohort of pediatric patients with esophageal eosinophilia. Hum Pathol..

[13] Zhao J (2011). 15-Lipoxygenase 1 interacts with phosphatidylethanolamine-binding protein to regulate MAPK signaling in human airway epithelial cells. Proc Natl Acad Sci USA.

[14] Walenga RW, Boone S, Stuart MJ (1987). Analysis of blood HETE levels by selected ion monitoring with ricinoleic acid as the internal standard. Prostaglandins..

[15] Mayatepek E, Lehmann WD (1996). 12- and 15-hydroxyeicosatetraenoic acid are excreted in the urine of peroxisome-deficient patients: evidence for peroxisomal metabolism *in vivo*. Pediatr Res..

[16] Feltenmark S (2008). Eoxins are proinflammatory arachidonic acid metabolites produced via the 15-lipoxygenase-1 pathway in human eosinophils and mast cells. Proc Natl Acad Sci USA.

[17] Chu HW (2002). Expression and activation of 15-lipoxygenase pathway in severe asthma: relationship to eosinophilic phenotype and collagen deposition. Clin Exp Allergy..

[18] Leenstra T (2006). T-helper-2 cytokine responses to Sj97 predict resistance to reinfection with Schistosoma japonicum. Infect Immun..

[19] Ariel A, Timor O (2013). Hanging in the balance: endogenous anti-inflammatory mechanisms in tissue repair and fibrosis. J Pathol..

[20] Uderhardt S, Krönke G (2012). 12/15-lipoxygenase during the regulation of inflammation, immunity, and self-tolerance. J Mol Med..

[21] Yamada T (2011). Eosinophils promote resolution of acute peritonitis by producing proresolving mediators in mice. FASEB J..

[22] Bocan TM (1998). A specific 15-lipoxygenase inhibitor limits the progression and monocyte-macrophage enrichment of hypercholesterolemia-induced atherosclerosis in the rabbit. Atherosclerosis..

[23] Wu M-Y (2012). Involvement of 15-lipoxygenase in the inflammatory arthritis. J Cell Biochem..

[24] Gulliksson M (2007). Expression of 15-lipoxygenase type-1 in human mast cells. Biochim Biophys Acta..

[25] Liu C (2009). 15-Lipoxygenase-1 induces expression and release of chemokines in cultured human lung epithelial cells. Am J Physiol Lung Cell Mol Physiol..

[26] Wei C, Zhu P, Shah SJ, Blair IA (2009). 15-oxo-Eicosatetraenoic acid, a metabolite of macrophage 15-hydroxyprostaglandin dehydrogenase that inhibits endothelial cell proliferation. Mol Pharmacol..

[27] Teitelbaum JE (2002). Eosinophilic esophagitis in children: immunopathological analysis and response to fluticasone propionate. Gastroenterology..

[28] Gupta SK, Fitzgerald JF, Kondratyuk T, HogenEsch H (2006). Cytokine expression in normal and inflamed esophageal mucosa: a study into the pathogenesis of allergic eosinophilic esophagitis. J Pediatr Gastroenterol Nutr..

[29] Wen Y (2007). The role of 12/15-lipoxygenase in the expression of interleukin-6 and tumor necrosis factor-alpha in macrophages. Endocrinology..

[30] Fairfax BP (2010). An integrated expression phenotype mapping approach defines common variants in LEP, ALOX15 and CAPNS1 associated with induction of IL-6. Hum Mol Genet..

[31] Zhang X (2012). Omeprazole blocks STAT6 binding to the eotaxin-3 promoter in eosinophilic esophagitis cells. Plos One.

[32] Bullock JZ (2007). Interplay of adaptive th2 immunity with eotaxin-3/c-C chemokine receptor 3 in eosinophilic esophagitis. J Pediatr Gastroenterol Nutr..

[33] Collins MH (2017). Newly developed and validated eosinophilic esophagitis histology scoring system and evidence that it outperforms peak eosinophil count for disease diagnosis and monitoring. Dis Esophagus..

[34] Conner JR, Kirsch R (2018). Editorial: validating reliability of the eosinophilic oesophagitis histological scoring system (EOE-HSS)-an important first step. Aliment Pharmacol Ther..

