# Supplementary material for: Utility of 15(S)-HETE as a Serological Marker for Eosinophilic Esophagitis
Source: Sci Rep. 2018 Sep 28;8:14498. doi: 10.1038/s41598-018-32944-8 (PMC6162315; doi:10.1038/s41598-018-32944-8)
Supplement: Supplementary file 1 — Supplemental tables [file 41598_2018_32944_MOESM1_ESM.docx]

Utility of 15(S)-HETE as a Serological Marker for Eosinophilic Esophagitis.

Shaolei Lu, MD, PhD [1,2]; Michael Herzlinger, MD [1,3]; Weibiao Cao, MD [2]; Lelia Noble, BA [2]; Dongfang Yang, MA [2]; Jason Shapiro, MD [3]; Jonathan Kurtis, MD, PhD [2]; Neal LeLeiko, MD, PhD [3]; Murray Resnick MD, PhD [2].

1) authors contributed equally

2) Department of Pathology and Laboratory Medicine, Rhode Island Hospital, Warren Alpert Medical School of Brown University.

3) Division of Pediatric Gastroenterology, Nutrition, and Liver Diseases, Hasbro Children’s Hospital, Warren Alpert Medical School of Brown University.

Corresponding author:

Shaolei Lu, M.D., Ph.D.

Pathologist and Assistant Professor

Director of Clinical Immunology Lab

Department of Pathology and Laboratory Medicine

Alpert Medical School of Brown University

Lifespan Academic Medical Center

593 Eddy Street, APC 12-122B

Providence, RI 02903

Tel: 401-444-7120

Fax: 401-444-4377

Pager: 401-350-8108

Email: slu@lifespan.org

**Supplemental table 1**. Peripheral 15(S)-HETE levels in patients of different PPI treatment duration at the time of endoscopy. Average dose = 1.4 mg/kg <30 kg body weight or 0.75 mg/day >30 kg body weight.

| Duration of PPI | N | Mean ±SEM  (pg/ml) | P-value  PPI ≤ 4 weeks vs. PPI > 4 weeks vs. no PPI | P-value  PPI vs. no PPI |
| --- | --- | --- | --- | --- |
| No PPI | 13 | 7023.5 ± 1988.3 | 0.6166* | 0.3287** |
| PPI treated | 18 | 11975.4 ± 3969.7 |  |  |
| PPI ≤ 4 weeks | 3 | 10707.3 ± 6466.3 |  |  |
| PPI > 4 weeks | 15 | 12229.1 ± 4662.6 |  |  |

* ANOVA analysis

** 2 tail t-test

**Supplemental table 2**. Peripheral 15(S)-HETE, AEC and cytokine levels in the EoE group subjects with and without atopy.

|  | EoE | | | Non-EoE | | |
| --- | --- | --- | --- | --- | --- | --- |
| Analyte* | With 2 or more atopies | With 1 or no atopy | P-value | With 2 or more atopies | With 1 or no atopy | P-value |
| HETE | 15613 ± 5482 | 5850 ± 2161 | 0.1198 | 6411 ± 1999 | 5319 ± 847 | 0.6312 |
| AEC | 0.63 ± 0.18 | 0.10 ± 0.05 | **0.0141** | 0.15 ± 0.06 | 0.18 ± 0.04 | 0.6962 |
| IL-4 | 648.3 ± 253.0 | 563.9 ± 404.8 | 0.8687 | 237.2 ± 103.2 | 224.3 ± 109 | 0.9328 |
| IL5 | 58.2 ± 19.1 | 63.1 ± 54.6 | 0.9387 | 10.9 ± 2.8 | 21.7 ± 14.5 | 0.4868 |
| IL-6 | 184.6 ± 64.1 | 363.2 ± 340.6 | 0.6547 | 35.5 ± 27.5 | 65.3 ± 37.8 | 0.5348 |
| IL-10 | 216.4 ± 61.1 | 293.2 ± 242.2 | 0.7848 | 62.0 ± 16.2 | 71.0 ± 46.7 | 0.8600 |
| IL-13 | 542.5 ± 184.6 | 440.2 ± 302.0 | 0.7880 | 224.0 ± 84.1 | 214.6 ± 97.4 | 0.9430 |
| IL-1 | 500.0 ± 154.0 | 623.0 ± 431.4 | 0.8087 | 258.8 ± 58.9 | 442.1 ± 147 | 0.9037 |
| IL-2 | 68.0 ± 45.4 | 42.8 ± 42.8 | 0.6980 | 3.47 ± 3.5 | 82.0 ± 27.3 | 0.3657 |
| IL-8 | 6.0 ± 3.3 | 15.2 ± 15.2 | 0.6075 | 1.72 ± 1.73 | 1.28 ± 0.89 | 0.8259 |
| IL-12 | 535.0 ± 296.6 | 474.2 ± 442.3 | 0.9145 | 107.8 ± 76.3 | 118.0 ± 68.5 | 0.9223 |
| TNF-α | 299.3 ± 88.6 | 291.8 ± 224.7 | 0.9775 | 135.6 ± 30.3 | 209.4 ± 69.8 | 0.9931 |
| IFN-γ | 1987 ± 822 | 2145 ± 1337 | 0.9252 | 672.1 ± 161.9 | 1906 ± 635 | 0.3498 |

* The unit of analytes is pg/ml except AEC which is K/µL. The values of analytes are presented as mean±SEM. P-values were calculated by 2-tail t-test.
